# Levels of parathyroid hormone and IGF binding protein 1 and associations with mortality and hip fractures in older women

**DOI:** 10.1038/s41598-024-80527-7

**Published:** 2024-11-26

**Authors:** Elin Uzunel, Hans Ranch Lundin, Ann-Charlotte Grahn Kronhed, Per Wändell, Helena Salminen

**Affiliations:** 1 Division of Family Medicine and Primary Care, Department of Neurobiology Care sciences and Society, Karolinska Institutet, Stockholm, Sweden; 2 Academic Primary Health Care Centre, Stockholm, Sweden; 3 Division of Prevention, Rehabilitation and Community Medicine, Department of Health, Medicine and Caring Sciences, Linköping University, Linköping, Sweden

**Keywords:** Osteoporosis, Hip fracture, All-cause mortality, Parathyroid hormone, IGFBP-1, Biomarkers, Medical research

## Abstract

In this study we examined the effect of simultaneously elevated levels of parathyroid hormone (PTH) (≥ 65 ng/mL) and high levels of insulin-like growth factor-binding protein 1 (IGFBP-1) on the 10-year risk of all-cause mortality and hip fractures. Blood tests for levels of PTH and IGFBP-1 was collected at baseline in 338 community-dwelling women in Stockholm aged between 69 and 79 years. Data on hip fractures and all-cause mortality over the next 10 years were retrieved from healthcare registers. The participants were divided into four groups depending on their levels of PTH and IGFBP-1: (A) normal PTH and low IGFBP-1; (B) normal PTH and high IGFBP-1; (C) elevated PTH and low IGFBP-1; (D) elevated PTH and high IGFBP-1. Group D was used as reference. Cox proportional hazard regression (HR) model was used to compare age-adjusted association with hip fractures and all-cause mortality of the four groups. The group with elevated levels of PTH and high IGFBP-1 had a two to three times higher risk of all-cause mortality compared to the other groups but we found no association with hip fractures.

## Introduction

Fragility fractures are associated with increased mortality, morbidity and impaired function^1,2^. They are especially common in postmenopausal women. Common sites of fragility fractures include the hip, distal forearm and vertebra. Osteoporosis is a skeletal disease characterized by decreased bone mass and deterioration of the micro-architecture of the bone, leading to increased bone fragility and greater risk of fractures. Bone mineral density can be measured, but other risk factors contribute to the individual risk of fracture^3–5^. Osteoporosis can also be caused by other diseases or medications.

Insulin-like growth factor-binding protein 1 (IGFBP-1) and parathyroid hormone (PTH) are both important actors in bone metabolism. They have been associated with metabolic syndrome and increased cardiovascular mortality, all-cause mortality and fracture risk^6–10^. Raised levels of PTH may be due to increased abnormal excretion from the parathyroid glands (primary hyperparathyroidism) or due to upregulation in response to vitamin D deficiency, kidney failure or malabsorption (secondary hyperparathyroidism)^11^. An increased level of PTH, which regulates calcium levels in the blood, is a marker for increased bone resorption. Elevated PTH has also been associated with cardiovascular mortality and all-cause mortality^6,10–15^. The insulin-like growth factor I (IGF-I) system, including IGF-I and IGFBPs 1–6, are important to the development of the skeleton and maintenance of bone mass. IGFBPs regulate the effect of IGF-I and seem to have effects of their own on bone and other tissues. The level of IGFBP-1 is negatively associated with bone mineral density^9,16–18^. Both low and high levels of IGFBP-1 have been associated with cardiovascular and all-cause mortality^19–21^. IGFBP-1 is also associated with insulin resistance, metabolic syndrome, diabetic and chronic kidney disease^7,22^.

As both PTH and IGFBP-1 levels are associated with an increased risk of fractures and mortality, each one separately, we wanted to investigate possible synergy between them. Earlier research on the same cohort has already revealed associations between levels of IGFBP-1 and osteoporosis, fracture risk and all-cause mortality^9,21^.

The aim of the present study was to investigate the effect of simultaneously elevated levels of PTH and IGFBP-1 on the risk of hip fractures and all-cause mortality after 10 years. We hypothesized that elevated levels of PTH (65 ng/L or more) might strengthen the association between high levels of IGFBP-1 and the 10-year risk of all-cause mortality and hip fractures.

## Methods

### Study population

**Fig. 1:**
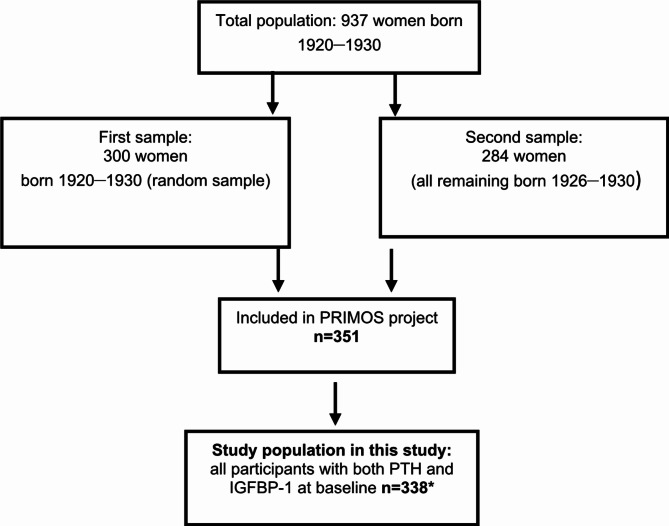
Flowchart of the study population. *One woman with a very high level of PTH was excluded.

This is a longitudinal, population-based cohort study. The cohort was initially gathered as part of a larger project called Primary Health Care and Osteoporosis (PRIMOS)^23–27^, which started in 1999 to investigate a population of older community-dwelling women. The inclusion criteria were that the participants should be between 69 and 79 years of age, living in a specific area of southern Stockholm (Bagarmossen) and able to visit the local primary healthcare centre for examination. In total, 937 women in this age group lived in the area at that time. First, a random sample of 300 women were sent letters inviting them to participate, of whom 179 accepted. Second, an invitation was sent to all 284 of the remaining women born between 1926 and 1930 (63 to 79 years of age), of whom 172 accepted. This gave a total of 351 participants out of 584 invited (60%). Frail health status or not being mobile enough to visit the primary healthcare centre were the most common reasons to decline participation in the study. Most of the participants (87.5%) were born in Sweden, 3.1% in Finland, 2.6% in Norway, 0.6% in Denmark and approximately 6% in non-Scandinavian countries. The participants were examined by the same physician from March 1999 until February 2001. Register data were collected from the Swedish Cause of Death Registry concerning mortality up to and including 31 December 2009. Blood samples for analyses, including PTH and IGFBP-1, were collected at baseline. After the exclusion of one subject with a very high level of PTH (775 ng/L), 338 subjects who had test results for both PTH and IGFBP-1were included in the present study (Fig. 1).

### Biochemical analyses

Fasting blood samples for biochemical analyses were collected at baseline and analysed on an ongoing basis or frozen at -70 °C until analysed. Levels of ionized calcium in serum (reference range 1.17–1.29 mmol/L), 25 hydroxy vitamin D in serum (nmol/L), intact PTH in plasma (reference range 10–65 ng/L), creatinine in plasma (reference range < 110 µmol/L for women and < 120 µmol/L for men) and albumin in plasma (reference range 37–48 g/L) were analysed routinely. Estimated glomerular filtration rate (GFR) was calculated by using the Cockcroft-Gault formula. A GFR < 60 ml/min/1.73 m^2^ was considered as renal failure since this cut-off has been used in previous studies of the elderly^28^. IGFBP-1 concentrations in serum were determined by a method using radioimmunoassay^29^.

.

### Variables and outcomes

**Fig. 2:**
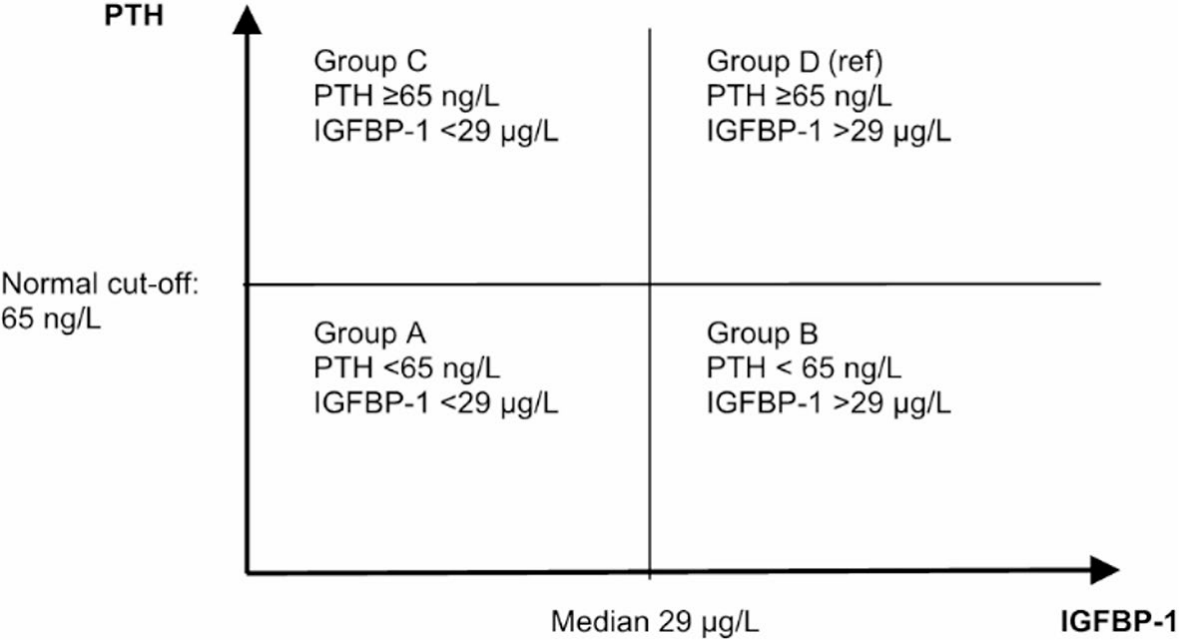
Division into groups A–D, based on levels of PTH and IGFBP-1.

During the study visit, data on ethnicity, age, medical history, current medication, lifestyle factors, time spent outdoors and participation in physical activities were registered or obtained from medical records. Height, weight, body mass index (BMI: kg/(m)^2^), bone mineral density (BMD) of the femoral neck and blood pressure were measured under standardised conditions. The outcome was all-cause mortality or hip fracture. The study time was set from the date of inclusion until 31 December 2009. Follow-up data was obtained from the Swedish Cause of Death Registry and data on inpatients and outpatients from the National Patient Register, both of which are kept by the National Board of Health and Welfare. The participants were divided into two subgroups of PTH: levels < 65 ng/L and levels ≥ 65 ng/L according to the normal reference interval that classifies values of ≥ 65 ng/L as elevated. The two groups with normal and elevated levels of PTH were each further divided according to their levels of IGFBP-1. We chose to divide by the median value of IGFBP-1 in this population, which was 29 µg/L. This resulted in four groups: (A) normal levels of PTH and low IGFBP-1; (B) normal levels of PTH and high IGFBP-1; (C) elevated levels of PTH and low IGFBP-1; and (D) elevated levels of PTH and high IGFBP-1, see Fig. 2.

### Statistical analyses

Baseline characteristics for continuous data were presented as mean (M), median (Md), and standard deviation (SD) and 95% confidence intervals (95% CI). Categorical data were presented as frequencies and percentages. For comparison of baseline characteristics between the groups we analysed the data with the Kruskal–Wallis test for skewed variables, and one-way ANOVA for normally distributed variables if Bartlett´s test showed that the variance was homogenous. For dichotomous variables, a Chi^2^-test was used if the frequency in any expected cell was five or more, otherwise Fisher’s exact test was used. Spearman’s correlation coefficient was used to explore whether there was any correlation between IGFBP-1 and PTH.

The Cox proportional hazards regression (HR) model was used for testing associations to all-cause mortality and hip fractures in the four groups (A–D). Tests for proportional hazards assumption were explored and the global test was insignificant. The covariates were tested for collinearity.

P-values < 0.05 were considered significant in the Cox proportional hazards regression model. When comparing differences between groups at baseline, P-values < 0.05 were considered significant. All analyses were performed with Stata statistical software version 14.2 (StataCorp. LLC, College Station, TX, USA).

## Results

We followed 338 community-dwelling women for about 10 years. Descriptive data is summarised in Table 1. The median age at baseline for all participants was 72.4 (IQR: 71.1–73.8). We compared the groups A–D, see Fig. 2. There were significant differences between the groups at baseline regarding age, BMI and BMD at femoral neck. Age was slightly higher in the two groups with elevated levels of PTH. Regarding BMI, the two groups with low levels of IGFBP-1 had higher BMI (no group had normal BMI). Femoral neck BMD was higher in the two groups with low levels of IGFBP-1 regardless of whether PTH levels were normal or high. Incidence of cardiovascular disease (ICD10 codes I00–I99), also differed between the groups. The groups with elevated PTH levels had higher incidence and the group with elevated levels of PTH and high levels of IGFBP-1 had the highest incidence of cardiovascular disease. The prescription of loop diuretics was highest in the group with elevated levels of PTH and high levels of IGFBP-1. The group with elevated levels of PTH and high levels of IGFBP-1 had the highest creatinine levels, hence they had the lowest GFR. Regardless of PTH levels, the two groups with high levels of IGFBP-1 had a GFR of < 60 ml/min/1.73 m^2^, i.e., below the limit for normal renal function. Albumin was slightly higher in the group with normal levels of PTH and low levels of IGFBP-1. There were no significant differences regarding a previous or present cancer diagnosis at inclusion, asthma/chronic obstructive pulmonary disease, diabetes, smoking, outdoor activities, prescription of bisphosphonates, insulin, oral antidiabetics, calcium/vitamin D supplements or plasma levels of ionized calcium. However, the number of people with ionized calcium above the upper reference limit (1.29 mmol/L) differed between the groups and was higher in the groups with elevated levels of PTH and highest in the group with elevated levels of PTH and low levels of IGFBP-1. The levels of 25-OH vitamin D was significantly lower in the groups with elevated PTH regardless of IGFBP-1 levels.

**Table 1:** Baseline characteristics of the four groups: (A) normal levels of PTH and low IGFBP-1; (B) normal levels of PTH and high IGFBP-1; (C) elevated levels of PTH and low IGFBP-1; (D) elevated levels of PTH and high IGFBP-1.

		All participants (*n* = 338)	Normal PTH < 65ng/L (*n* = 250)		Elevated PTH ≥ 65ng/L (*n* = 88)		*p*-value
			Low IGFBP-1*n* = 124	High IGFBP-1*n* = 126	Low IGFBP-1*n* = 43	High IGFBP-1*n* = 45	
PTH^a^ ng/L	Md (min-max)	52.9 (13.7- 171.8)	48 (21–65)	45 (14–65)	76 (66–157)	83 (66–172)	
IGFBP-1^b^ µg/L		29 (5-169)	19 (5–28)	43 (29–104)	20 (8–28)	43 (29–169)	
Age (y), Md (IQR)		72.4 (71.1–73.8)	72.2 (71.1–73.4)	72.2 (71.1–73.7)	73.0 (71.2–74.2)	73.2 (71.8–76.1)	0.027^o*^
BMI^c^ kg/m^2^, mean (SD)		27 (4)	28 (4)	25 (3)	30 (5)	27 (5)	< 0.001^o*^
BMD^d^ at femoral neck, mean (SD)		0.65 (0.10)^k^	0.68^l^ (0.10)	0.62^l^ (0.10)	0.67^l^ (0.08)	0.63 (0.08)	< 0.001^p*^
Diseases n (%)							
Earlier/present cancer		44 (13)	13 (10)	20 (16)	3 (7)	8 (18)	0.281^q^
Asthma/COPD^e^		35 (10)	13 (10)	11 (9)	8 (19)	3 (7)	0.288^q^
CVD^f^		126 (37)	39 (31)	35 (28)	25 (58)	27 (60)	< 0.001^r*^
Diabetes (%)		28 (8)	9 (7)	10 (8)	7 (16)	2 (4)	0.234^q^
Risk factors n (%)							
Cigarette smoking		53 (16)	21 (17)	22 (17)	5 (12)	5 (11)	0.638^r^
Outdoors ≥ 30 min/day		286 (85)^l^	106 (85)	112 (89)	36 (84)	32 (73)^l^	0.082^r^
Medications n (%)							
Loop diuretics		28 (8)	7 (6)	4 (3)	6 (14)	11 (24)	< 0.001^q*^
Bisphosphonates		8 (2)	3 (2)	5 (4)	0 (0)	0 (0)	0.474^q^
Calcium + VitD_3_		26 (8)	13 (10)	10 (8)	1 (2)	2 (4)	0.316^q^
Insulin		6 (2)	2 (2)	3 (2)	0 (0)	1 (2)	0.941^q^
Oral Anti diabetics		11 (3)	5 (4)	5 (4)	1 (2)	0 (0)	0.694^q^
Blood levels mean							
Calcium mmol/L^g^ (SD)		1.2 (0.04)^l^	1.2 (0.04)	1.2 (0.03)^l^	1.2 (0.06)	1.2 (0.05)	0.505^o^
Hypercalcemia n (%)^h^		11 (3)^l^	3 (2)	0 (0)^l^	5 (12)	3 (7)	0.001^q*^
Creatinine µmol/L^i^ (SD)		85.3 (10.6)^m^	84.4 (9.9) ^l^	83.8 (9.0) ^l^	85.0 (10.4)	92.4 (13.7)	0.001^o*^
Albumin g/L^i^ (SD)		39.7 (2.9)^m^	40.3 (2.9) ^m^	39.4 (3.0)	39.1 (2.4)	39.2 (2.6)	0.020^p*^
25-OH vitamin D, nmol/L (IQR)		92 (70 —111)	96 (77—110)^m^	93 (72—115)^n^	76 (61—101)	79 (70—102)	0.0045^o^
GFR (SD)^j^		58.1 (12.0)^m^	61.2 (10.8) ^l^	55.0 (11.0) ^l^	64.0 (13.0)	52.5 (12.3)	< 0.001^p*^
GFR < 60^j^ yes n (%)		195 (58)	58 (47)	86 (68)	17 (40)	34 (76)	< 0.001^r^

^a^ PTH = parathyroid hormone, ^b^ IGFBP-1 = Insulin-like growth factor binding protein 1, ^c^ BMI = Body mass index, ^d^ BMD = bone mineral density, g/cm^2^, ^e^ COPD = chronic obstructive pulmonary disease, ^f^ CVD = cardiovascular disease (defined as ICD10 codes I00–I99), ^g^ ionized calcium in plasma, ^h^ >1.29 mmol/L, ^i^ serum, ^j^ GFR = glomerular filtration rate (Cockcroft-Gault), (mL/min/1.73 m^2^), ^k^ six missing values, ^l^ one missing value, ^m^ two missing values, ^n^ three missing values, ^o^ Kruskal–Wallis, ^p^ One-way ANOVA ^q^ Fisher’s exact test.

^r^ Chi2 test, ^*^P-value less than 0.05.

We used the Cox proportional hazard regression (HR) model to compare the age-adjusted association with all-cause mortality between the groups (A–D). We found that the mortality rate in the group with elevated levels of PTH and high levels of IGFBP-1 was two to three times higher than in the other groups. The result was similar when we excluded the participants with diabetes at inclusion. When we compared the age-adjusted association of elevated levels of PTH compared to normal levels of PTH on all-cause mortality, the risk was almost two times higher in the group with elevated levels of PTH. There was no significant difference in age-adjusted all-cause mortality when we compared low levels to high levels of IGFBP-1 (Table 2).

**Table 2:** Association with age-adjusted all-cause mortality by Cox proportional hazard regression model: crude associations comparing elevated levels vs. low/normal levels of PTH and IGFBP-1 and comparison of the group with elevated levels of PTH and high IGFBP-1 with the other groups.

Outcome: all-cause mortality	Reference	HR (95% CI)	*p*-value
Elevated PTH (≥ 65 ng/L)	Normal PTH (< 65 ng/L)	1.89 (1.15–3.08)	0.011
High IGFBP-1(≥ 29 µg/L)	Low IGFBP-1 (≤ 28 µg/L)	1.39 (0.86–2.24)	0.179
Elevated PTH High IGFBP-1	Normal PTH Low IGFBP-1	2.83 (1.48–5.41)	0.002
Elevated PTH High IGFBP-1	Normal PTH, High IGFBP-1	2.64 (1.40–4.99)	0.003
Elevated PTH High IGFBP-1	Elevated PTH Low IGFBP-1	2.30 (1.03–5.14)	0.043

We also compared age-adjusted cardiovascular mortality in the group with elevated levels of PTH and high IGFBP-1 with the other groups (Cox proportional hazard regression model) and obtained following results: HR 3.93, *p* = 0.001 (compared to normal PTH and low IGFBP-1); HR 2.85, *p* = 0.004 (compared to normal PTH and high IGFBP-1); HR 2.48, *p* = 0.049 (compared to elevated PTH and low IGFBP-1). There was no significant difference in the age-adjusted HR of hip fracture when we compared the group with elevated levels of PTH and high IGFBP-1 with the other groups.

IGFBP-1 was not correlated to PTH using Spearman´s correlation coefficient r_s_=-0.01 (*p* = 0.823).

Of the 338 participants, 69 women (20%) died and 38 (11%) suffered a hip fracture during the ten-year follow-up period. The group with elevated levels of PTH and high levels IGFBP-1 were overrepresented among the 69 deaths. We found no statistical difference between the groups regarding hip fractures, but the group with normal levels of PTH and low levels IGFBP-1 had the smallest percentage of hip fractures, see Table 3.

**Table 3:** Number and percentage of deaths (all-cause mortality) and hip fractures within the groups during the ten-year follow-up period.

	Normal PTH^a^ *n* = 250		Elevated PTH^a^ *n* = 88		*P*-value^c^
	Low IGFBP-1^b^ *n* = 124	High IGFBP-1^b^ *n* = 126	Low IGFBP-1^b^ *n* = 43	High IGFBP-1^b^ *n* = 45	
Died n (%)	20 (16)	22 (17)	9 (21)	18 (40)	0.005
Hip fracture n (%)	10 (8)	16 (13)	6 (14)	6 (13)	0.565

^a^PTH= parathyroid hormone, ^b^Insulin-like growth factor binding protein 1, ^c^Chi^2^test, IGFBP-1 = Insulin-like growth factor binding protein 1.

## Discussion

We wanted to explore the association between synchronous elevated levels of PTH and high levels of IGFBP-1 and the 10-year risk of all-cause mortality and hip fractures. The most striking result of this study is that simultaneously elevated levels of PTH and high levels of IGFBP-1 seem to result in a higher all-cause mortality rate than elevated levels of PTH and low levels of IGFBP-1, and normal levels of PTH regardless of whether levels of IGFBP-1 are high or low. When we compared elevated levels of PTH to normal levels of PTH, we also found an increased risk of all-cause mortality, although not as great. In a previous study, we presented results from the same cohort showing that both high and low IGFBP-1 levels (compared to moderate levels) were associated with an increased risk of all-cause mortality, and that there was a linear relationship between IGFBP-1 levels and fragility fractures^9,21^. We therefore expected to find an association with hip fractures as well, but we found no correlation to the 10-year risk of hip fractures.

To the best of our knowledge, the synchronous effect of levels of IGFBP-1 and PTH have not previously been studied. The kidney function differed between the groups that we compared, and it is worth to consider that this may influence the results.

Other research has also found an association with either low levels of IGFBP-1 and cardiovascular mortality/risk factors in elderly men and women^20,30^ or high levels of IGFBP-1 and all-cause mortality in elderly men and women^31^. Higher levels of IGFBP-1 have been observed in patients with osteoporosis compared with non-osteoporotic controls (a study of both men and women, with and without fractures)^18^. Pye et al. found that higher levels of IGFBP-1 (adjusted for age, centre and BMI) were associated with calcaneal BMD in men, but since this association became non-significant after further adjustment for lifestyle factors, sex steroids, sex-hormone binding globulin (SHBG) and PTH levels, they considered that this association could be explained by other factors. They found no association between levels of IGFBP-1 and self-reported fractures^32^. Another study (both men and women) found that levels of IGFBP-1 were not significantly associated with BMD at the hip or spine after adjusting for age and BMI^33^. In a study focusing on age-related bone loss by comparing child and parent (without osteoporosis), Lee et al. found a significant difference in BMD at the femoral neck between the younger and older member of the family pairs, but no difference in levels of IGFBP-1^34^. So, there are conflicting results from earlier studies regarding the correlation between levels of IGFBP-1 and BMD/fragility fractures. While we previously found a linear association in the same cohort between levels of IGFBP-1 and hip fractures, this was not apparent in this study when we combined levels of IGFBP-1 and PTH. This could be because the addition of PTH to the equation weakens the association, contradicting our hypothesis, or that in this study we compared IGFBP-1 levels at a group level in contrast to the linear association we found earlier. Studies of correlations between levels of PTH and all-cause and cardiovascular mortality have also shown varying results, although according to a meta-analysis by Yang et al., the inconsistent findings may be due to the fact that different populations have been analysed. Indeed, they concluded that elevated PTH levels are an independent predictor of all-cause mortality^35^. This may be supported by the fact that studies conducted in general in older populations found an increased all-cause mortality^15,36^. Interestingly, Hagström et al., demonstrated that elevated PTH levels (> 50 ng/L) were associated with higher cardiovascular mortality, even if they were within the normal range (< 65 ng/L)^13^.

An elevated level of PTH (as in hyperparathyroidism) is often associated with a higher risk of fracture, as it leads to increased bone resorption^11^. Elevated levels of PTH in combination with low serum 25-hydroxyvitamin D, even without secondary hyperparathyroidism, have also been associated with both increased bone turnover and mortality^10,12^. Sambrook et al., showed that an elevated level of PTH was a predictor of mortality independent of 25-hydroxyvitamin D levels and calcaneal bone mass (measured by ultrasound, calcaneal BUA)^15^. While we found a correlation to all-cause mortality, it is worth noting that the incidence of cardiovascular disease was higher in the groups with elevated PTH levels and highest in the group with elevated levels of PTH and high levels of IGFBP-1 (60%). The association with age-adjusted cardiovascular mortality by Cox proportional hazard regression model was also significant comparing the groups (A–D), and had higher HRs than the association with age-adjusted all-cause mortality: HR 3.93 compared to 2.83, 2.85 compared to 2.64 and HR 2.48 compared to 2.30. This may indicate that CVD explains a large part of the mortality in this study. It is also interesting that we found that elevated high levels of PTH were associated with an increased 10-year risk of all-cause mortality, but that high levels of IGFBP-1 seemed to worsen the scenario further.

## Strengths and limitations

The longitudinal design with a follow-up period of around 10 years is a strength. While the number of participants was relatively large, the low number of fractures that occurred (*n* = 38), may be too small to identify significant associations between PTH and IGFBP-1 levels and hip fractures. Sweden has comprehensive registers that made it possible to retrieve outcome data on all of our subjects for follow-up purposes. Limitations of the study may include the lack of data on secondary hyperparathyroidism in the cohort. Another limitation is that the cohort consisted solely of women, meaning we are unable to draw any conclusion about men. On the other hand, this kind of association may well differ between the sexes. Also, the fact that frail health status or not being mobile enough to visit a primary healthcare centre were reasons to exclude prospective participants could indicate that our sample of older women might be healthier than women of this age group in general. One more limitation is that there is no normal range set for IGFBP-1 for this analysis method. In our material there was a median value of 29, (range 5–169, IQR 20– 43). This is very much in line with earlier studies of postmenopausal women in Sweden (also a method that, according to Popova et al.^29^), one of which showed a geometric mean of 34.9 µg/L, range 8–109 µg/L, and another a median value of 37 µg/L, IQR 28–49^37,38^). We divided the material into two groups based on the median value of our population.

## Conclusion

Elevated levels of PTH did not strengthen the association between high IGFBP-1 levels and hip fractures in older women. However, elevated levels of PTH and IGFBP-1 were indeed associated with a higher 10-year risk of all-cause mortality. Osteoporotic fractures have a major impact on mortality, function and quality of life. Because the aetiology of osteoporosis is complex, there may be unexplored variables/factors that could help identify people at risk of fractures. The role of IGFBP-1 is still unclear and needs to be explored further.

## Data Availability

The datasets used during the current study are available from the corresponding author on reasonable request.
